# Exploring the genetic profiles linked to senescence in thyroid tumors: insights on predicting disease progression and immune responses

**DOI:** 10.3389/fonc.2025.1545656

**Published:** 2025-02-06

**Authors:** Baoliang Zhang, Yanping Pang

**Affiliations:** ^1^ Department of Emergency, Tongji Hospital of Tongji University, Shanghai, China; ^2^ Department of Ultrasound, Tongji Hospital of Tongji University, Shanghai, China

**Keywords:** thyroid cancer, cellular senescence, least absolute shrinkage and selection operator, tumor immune microenvironment, prognosis

## Abstract

**Introduction:**

Thyroid cancer (THCA) is the most common endocrine tumor. Research on Cell Senescence Associated Genes (CSAGs), which impact many cancers, remains limited in the THCA field.

**Methods:**

In this study, we downloaded THCA sample data from several public databases and selected a set of CSAGs for subsequent analysis. Differential expression genes (DEGs) obtained through differential analysis were intersected with prognostic genes identified by Cox regression analysis to explore the correlation among these crossed genes. We constructed a prognostic model using the Least Absolute Shrinkage and Selection Operator (LASSO) algorithm and verified its efficacy. Kaplan-Meier survival curves were plotted, and Receiver Operating Characteristic (ROC) curves rigorously confirmed the accuracy of model predictions.

**Results:**

To evaluate the predictive power of prognostic models across different phenotypic traits, we performed survival analysis, Gene Set Enrichment Analysis (GSEA), and immune-related differential analysis. Differences in tumor mutation burden (TMB) and treatment response between high-risk and low-risk patient groups were also analyzed. Finally, the predictive effect of our model on immunotherapy response was validated, showing promising results for THCA patients.

**Discussion:**

Our study enhances the understanding of THCA cell senescence and provides new therapeutic insights. The proposed model not only accurately predicts patient survival but also reveals factors related to immunotherapy response, offering new perspectives for personalized medicine.

## 1 Introduction

Accounting for 3-4% of all cancers, thyroid cancer (abbreviated as THCA or TC) holds the position of being the endocrine tumor that occurs most frequently (1). Over the past few decades, there has been a consistent rise in its incidence, with some studies suggesting this may be related to the rising incidence of differentiated thyroid cancer (DTC) (2, 3). Compared to 40 years ago, the detection rate of THCA has increased by more than 400%, with the rise in diagnoses of small, indolent papillary thyroid carcinomas (PTCs) likely contributing to the overall increase in THCA incidence (4). Globally, the incidence of THCA is influenced by geographic location, with higher rates observed in high-income countries and certain island nations (5). The origin of THCA can be traced back to either the follicular epithelial cells or the parafollicular cells, alternatively referred to as C cells, within the thyroid gland. Based on the tumor’s origin and its level of differentiation, it encompasses various subtypes: PTC, which is the most prevalent, follicular thyroid carcinoma (FTC), thyroid oncocytic carcinoma (OCA, previously termed Hürthle cell thyroid carcinoma), differentiated high-grade thyroid cancer (DHGTC), poorly differentiated thyroid carcinoma (PDTC), anaplastic thyroid cancer (ATC), and medullary thyroid carcinoma (MTC). Clinically, PTC, FTC (6), OCA, and DHGTC are collectively referred to as DTC, which accounts for more than 90% of all THCA cases, making it the most common subtype of thyroid cancer (7). MTC accounts for only 1-2% of THCA cases (5). DHGTC, PDTC, and ATC are all of follicular epithelial cell origin, while MTC originates from parafollicular cells (7). Due to the asymptomatic nature of THCA, it is difficult to detect early in clinical practice. Approximately half of cases are not suspected or detected until other diagnostic procedures or thyroid-related surgeries are performed (8). Despite the generally favorable prognosis for the majority of THCA patients, with certain studies reporting a 5-year relative survival rate surpassing 90% for those with localized disease, 10-15% of THCA patients will experience disease recurrence. Approximately 5% of patients will have distant metastasis to organs such as in the instance of the lungs and bones, and occasionally, cancer-specific mortality may occur (9). Furthermore, not all THCA patients have a good prognosis. The survival rate for patients with distant metastasis varies by pathological subtype (10). The survival rate after 10 years stands at roughly 45% for patients with metastatic DTC, whereas for those with MTC, it drops to approximately 20%. ATC has an exceptionally grim prognosis, characterized by a median survival duration of merely 3 to 6 months (11).Currently, the primary options for treating THCA include thyroid surgery, therapy with radioactive iodine, and TSH suppression. Surgery remains the preferred initial treatment when criteria for resection are met. Postoperative radioactive iodine therapy or observation as standard care is effective for most DTC patients, however, for a specific group of patients, its effectiveness is constrained. For progressive or symptomatic DTC and MTC patients, although existing targeted therapies can extend progression-free survival (PFS), they do not provide a cure (12). Conventional treatments such as radioactive iodine ablation and chemotherapy are ineffective for highly invasive and fatal ATC (9). Additionally, studies have suggested that PD-L1-targeted immunotherapy may prolong disease-free survival (DFS) and could potentially become an effective treatment option for advanced THCA (13). In summary, early diagnosis and effective treatment of THCA remain significant challenges, necessitating continued exploration of new therapeutic targets.

Cellular senescence-associated genes (CSAG) refer to a cell state triggered by various physiological processes. Among the various factors contributing to this state are DNA damage, malfunctioning telomeres, the activation of oncogenes, mitochondrial dysfunction, as well as oxidative stress, and others (14). Senescent cells exhibit numerous characteristics, such as alterations in chromatin and secretory proteins, increased expression of senescence markers, immune evasion (15), loss of proliferative capacity, and secretion of inflammatory cytokines, chemokines, and growth factors (16). The intricate secretory proteins produced during the process are collectively referred to as the senescence-associated secretory phenotype (SASP). The International CSAG Association has proposed a consensus defining the phenotype of senescent cells based on four key features: cell cycle withdrawal, macromolecular damage, SASP, and metabolic dysregulation (17). Cell cycle inhibitors (CKIs) (14), p27KIP1 (18), p21CIP1 (CDKN1A), and Cyclin-dependent kinase inhibitor 2A (p16INK4A, CDKN2A) can participate in the CSAG process by regulating the cell cycle. For instance, upregulation of CDKN1A and CDKN2A can lead to hypophosphorylation of the retinoblastoma protein, thereby inhibiting E2F transcriptional activation and causing cell cycle arrest (19). Macromolecular damage, such as DNA, protein, and lipid damage, can also contribute to the CSAG process through activation of the tumor suppressor pathways involving p53/p21CIP1 and p16INK4A/RB (20). SASP is a complex secretory process that includes hundreds of different proteins and non-protein molecules. The full composition of SASP remains incompletely defined, but common molecules include interleukins such as IL-1α, IL-1β, IL-6, chemokines such as CXCR2 and CCL2, and growth factors like IGFBP7 (21). Studies indicate that AMP-activated protein kinase (AMPK), a kinase activated by the ratios of AMP: ATP and ADP: ATP during the CSAG process, has a function in modulating the cellular cycle (17). The physiological processes associated with CSAG play crucial roles in normal human development and are closely related to biological processes such as cancer therapy and tissue repair (16, 22). In cancer, the SASP secreted during CSAG can alter the tumor microenvironment (TME), induce immune surveillance of precancerous cells, and suppress cancer progression (14, 23). However, the persistent DNA damage and inflammatory factors generated by the senescence process may also promote tumor development and angiogenesis (24). In THCA, studies have indicated that the B-RafV600E mutation may participate in the senescence process of PTC cells by upregulating dual-specificity phosphatases (DUSPs) (25). Nevertheless, the role of CSAG in THCA remains insufficiently explored, different subtypes of thyroid cancer may respond differently to CSAG, highlighting the need for further investigation into its specific applications and interpretations in thyroid cancer research, as well as the correlation between CSAG and gender.

In this study, we not only downloaded THCA sample data from multiple public databases, but also selected a set of cellular senescence-associated genes (CSAGs) for subsequent analysis. Differentially expressed genes (DEGs) obtained through differential expression analysis were intersected with prognostic genes identified via Cox regression analysis, and the correlation among the intersecting genes was further investigated. Based on this, we employed the Least Absolute Shrinkage and Selection Operator (LASSO) algorithm both for finalizing the selection of model genes and for constructing the prognosis model. The precision of the predictions made by the model was rigorously confirmed using both Kaplan-Meier (KM) survival curves and Receiver Operating Characteristic (ROC) analysis. To evaluate the predictive capability of the prognostic model across different phenotypic characteristics, we subsequently conducted a thorough analysis comparing the risk groups. Besides performing survival analysis and Gene Set Enrichment Analysis (GSEA), we also carried out immune-related differential analyses that centered on the expression patterns of immune regulators, tumor-associated immune cells, and immune checkpoints. We examined variations in tumor mutational burden (TMB) and treatment responses between patient groups categorized as high- and low-risk. Additionally, we performed stratified KM survival analysis based on risk scores, giving special attention to immune checkpoints and TMB.

## 2 Material and methods

### 2.1 Data acquisition and preprocessing

Initially, we utilized the R package named “TCGAbiolinks” to obtain RNA sequencing data, comprehensive clinical details, and mutation information pertaining to THCA patients, sourced from the Cancer Genome Atlas (TCGA) database, which can be accessed at https://portal.gdc.cancer.gov. For the aim of facilitating better gene differential expression analysis between samples, the transcriptomic data was transformed into Transcripts Per Million (TPM) format. By employing the “GEOquery” package, we acquired transcriptomic data along with the corresponding clinical information for THCA patients (GSE84437) from the Gene Expression Omnibus (GEO) database, accessible at http://www.ncbi.nlm.nih.gov/geo. The cohort that underwent immunotherapy, known as IMvigor210, was downloaded through the R package “IMvigor210CoreBiologies”. The list of age-related genes used in this paper were all obtained from previous literature summary (**Supplementary Table 1**). In addition, we included CSAG in the list and extracted intracellular gene expression levels from TCGA samples. The open-source databases involved in this study have no restrictions on data acquisition and use, and no additional ethical approval is required. All analytical procedures in this study strictly adhere to ethical guidelines.

### 2.2 Constructing and validating the predictive model for prognosis

Differential expression analysis of CSAG between normal and tumor tissues was conducted using the “limma” package, with the results of the DEGs being graphically represented through a volcano plot. Our criterion for DEGs selection was set as |logFC|> 0.585, and the adjusted p value was <0.05. Subsequently, we applied univariate Cox regression analysis to ascertain CSAGs that hold prognostic importance, and the resulting prognostic genes were displayed in a forest plot. A Venn diagram was utilized to illustrate the overlapping DEGs and prognostic genes, while an analysis was performed to investigate the relationships among these prognostic genes that were differentially expressed. Outcomes of this correlation analysis were displayed in a circular correlation plot. Afterwards, the TCGA cohort formed the training dataset, and the GSE84437 cohort was assigned for validation. LASSO is a regression analysis method, which can simplify the model and improve the prediction accuracy by introducing a penalty term to achieve both variable selection and model parameter estimation. In bioinformatics, the advantage of LASSO is that it can effectively process high-dimensional data to screen out features or genes that have a significant impact on response variables, so as to assist in disease diagnosis, drug target discovery and other studies. The parameter standard for LASSO is “cvfit$lambda.min”. Utilizing the LASSO algorithm, a prognostic prediction model was built within the training set. The source of gene list input in LASSO model was differentially expressed prognostic genes. The model’s predictive outcomes were quantified as risk scores, which were derived by summing up the products of the levels of expression for each gene multiplied by its respective coefficient, as the formula presented below:


$$\text{Risk score}=\sum_{i=1}^{n}\left[Exp_{gene_{i}}*\beta_{i}\right]$$

The level of expression for each gene in the model is denoted as Exp_genei_, with β_i_ representing the gene coefficient. The selection of model genes is determined by the optimal λ value, and the variation of coefficients across different genes with respect to log(λ) is illustrated in the coefficient distribution plot. The λ value that yields the lowest partial likelihood deviance is taken as the optimal one. Subsequently, we perform the following analyses on both training and validation datasets, applying the same procedure to two independent cohorts. Within each cohort, using the median risk score as a benchmark, patients are grouped into high-risk and low-risk categories. The “survival” and “survminer” packages were then used for KM analysis to visually show the difference in overall survival (OS) of different risk groups over time. In order to assess the model’s predictive capabilities, the survival probabilities for 1-year, 3-year, and 5-year durations are depicted via ROC curves, and the model’s prognostic accuracy is assessed using the area under the curve (AUC) as a metric. AUC > 0.5 proves that the model has good testing efficiency.

### 2.3 Prognostic and enrichment analysis for different risk groups

We standardized the expression profiles of model genes and compared them between the two groups across both datasets. A risk curve was generated by ordering individual samples in ascending order of their risk scores, and the variation in survival time as the risk score increased was analyzed. Furthermore, within the TCGA cohort, after stratifying the patients into either Stage I-II or Stage III-IV, we performed survival analysis on them. To assess prognostic differences across various tumor stages, we utilized KM curves to compare high-risk and low-risk groups, in order to explore the impact of tumor stage on the model’s predictive outcomes.

Subsequently, using pathways obtained from the MEDICUS module of the Kyoto Encyclopedia of Genes and Genomes (KEGG) database, we applied GSEA to identify functional pathways with differential distribution between the two groups and subsequently represented the findings visually. Functional pathways that exhibited an enrichment score above 0 were interpreted as having gene expression upregulated in the high-risk cohort, whereas those showing a score below 0 implied upregulation in the low-risk cohort.

### 2.4 Analysis of differential immune features

We conducted an immune regulatory expression profiling analysis, including five categories of immune regulatory molecules: chemokines, growth factors and regulators, soluble or shed receptors/ligands, and interleukins. Heatmaps were utilized to visualize the disparities in expression between the high-risk and low-risk groups. The following six algorithms utilized for TME deconvolution: CIBERSORT, CIBERSORT ABS, EPIC, MCP-counter, quanTIseq, TIMER, and xCell, were implemented using R packages. Utilizing these algorithms, we conducted a thorough examination of the relationships between the model genes and the degrees of immune cell infiltration, and then portrayed the findings of these associations through various heatmaps.

Additionally, we carried out a comprehensive examination of immune checkpoints to explore potential immune therapy targets relevant to THCA. The gene expression levels of 31 selected immune checkpoints were compared between the two patient groups. Within each group, patients were categorized into two subgroups, based on whether their expression values for the immune checkpoint molecules exceeded or fell below the median value, followed by KM survival analysis to assess the survival probability differences across the four subgroups. This procedure was performed independently for each immune checkpoint molecule, resulting in 31 survival curve plots.

### 2.5 Mutation analysis and survival analysis of TMB

By sourcing mutation data from the TCGA database, we conducted computations and comparisons of the TMB between two patient groups, and subsequently visualized the disparities through the use of box plots. To gain a deeper insight into how risk scores correlate with TMB, we carried out a Pearson correlation analysis and developed scatter plots to provide a clear visual representation of the findings. Additionally, we divided the TCGA samples into two subsets using the median TMB value as the threshold: high-TMB (H-TMB) and low-TMB (L-TMB). KM survival curves were then plotted to clearly illustrate the survival differences between these two groups. In order to determine the joint impact of TMB and risk scores on survival outcomes, patients were divided into four distinct categories, each representing a unique combination of their TMB and risk level: the high TMB-high risk group, the high TMB-low risk group, the low TMB-high risk group, and the low TMB-low risk group. Survival differences among these four subgroups were also visualized using KM survival curves.

### 2.6 Predictive role of the model in immunotherapy response

We obtained immune phenotype score (IPS) data for TCGA samples from The Cancer Immunome Atlas (TCIA, https://tcia.at/). By examining patient responses to anti-CTLA-4 and anti-PD-1 antibodies, the IPS was categorized into four distinct groups: those negative for both anti-CTLA-4 and anti-PD-1 (ips_ctla4_neg_pd1_neg), negative for anti-CTLA-4 but positive for anti-PD-1 (ips_ctla4_neg_pd1_pos), positive for anti-CTLA-4 but negative for anti-PD-1 (ips_ctla4_pos_pd1_neg), and positive for both (ips_ctla4_pos_pd1_pos). Following this classification, a comparative analysis was undertaken to explore the varying responses of high-risk and low-risk groups to different immune checkpoint inhibitor treatment strategies. A violin plot was generated to visualize these results. Next, we validated the robustness of the model prognostic predictions using the IMvigor210 immunotherapy cohort using the”IMvigor210CoreBiologies” packages. After applying the prognostic model to the IMvigor210 cohort, utilizing the median risk score as a cutoff, the samples were categorized into two distinct groups: those belonging to the high-risk category and those in the low-risk category. A survival analysis was then carried out for these groups, with the results being graphically represented using KM survival curves. The outcomes of chemotherapy were classified into four categories: complete response (CR), partial response (PR), progressive disease (PD), and stable disease (SD). These categories were then simplified into two binary groups: CR/PR versus SD/PD. Within this setup, a comparison was made of the risk scores belonging to the two patient groups. Additionally, we selected 48 immune checkpoint molecules for further investigation. The IMvigor210 cohort’s patients, within each risk group, were additionally subclassified into high and low subgroups, according to the expression levels exhibited by the chosen checkpoint molecules. Thus, for each immune checkpoint molecule, patients were grouped into four subgroups. To identify immune checkpoints that are significantly correlated with survival outcomes, we conducted another KM survival curve analysis to assess the prognostic differences among these subgroups.

### 2.7 Statistical analysis

Depending on the distribution of the data, we evaluated the relationships among variables by utilizing either Pearson or Spearman correlation coefficients. When continuous variables met the normality assumption, a t-test was applied to compare paired samples; otherwise, the Mann-Whitney U test was used for those that did not conform to normality. Based on the situation, either the Chi-square test or Fisher’s exact test was utilized for making comparisons among categorical variables. For the prognostic assessment of categorical variables, survival curves were generated through the KM method, and statistical significance was evaluated using the log-rank test. Statistical significance was established at a p-value below 0.05, denoted as follows: * indicates p < 0.05, ** for p < 0.01, *** for p < 0.001, and **** for p < 0.0001. R software, specifically version 4.1.3, was utilized to carry out all statistical analyses. Unless mentioned otherwise, the “ggplot2” package was used to produce the graphs.

## 3 Results

### 3.1 Constructing and validating the predictive model for prognosis

**Figure 1:**
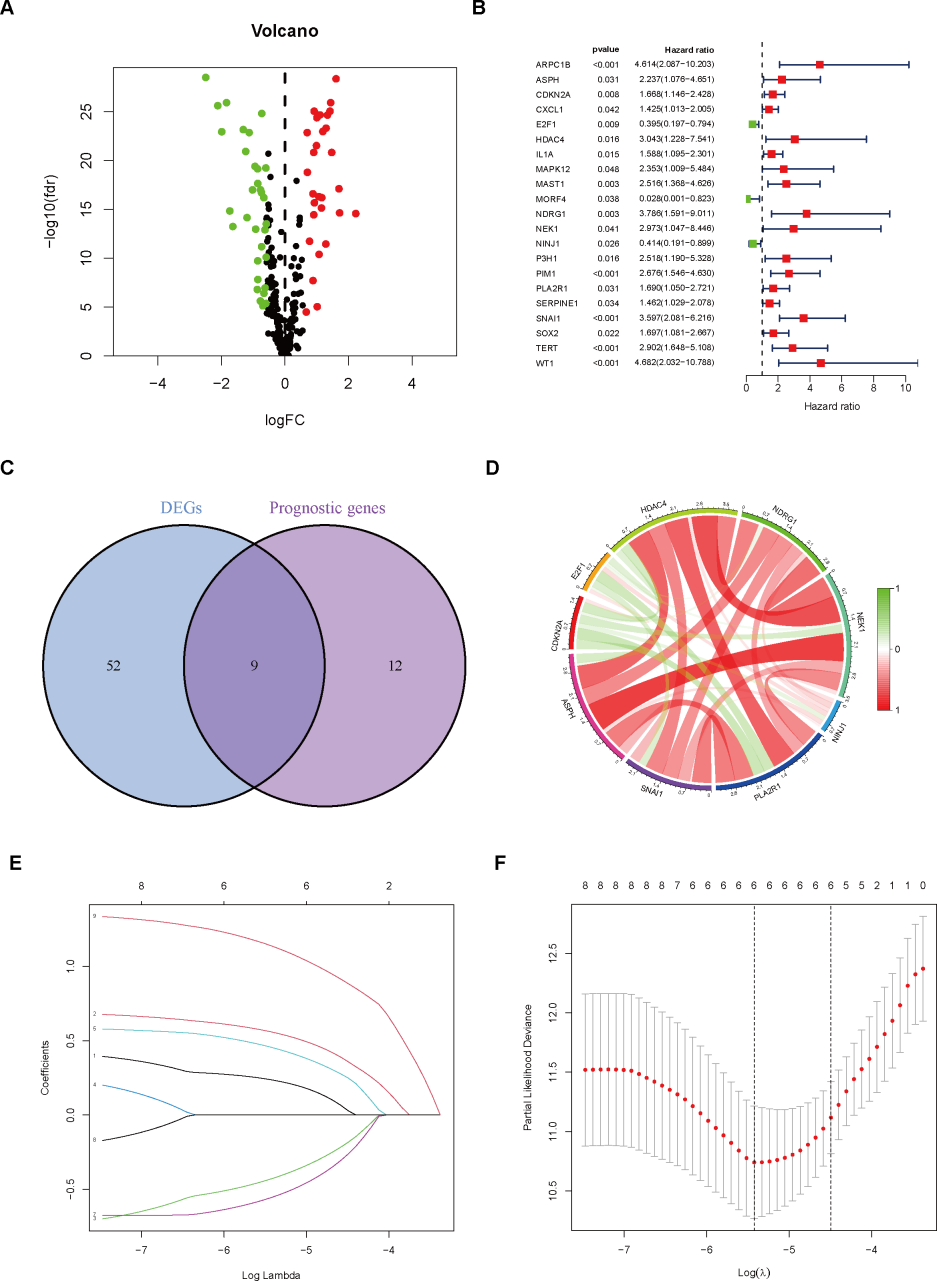
Gene Selection and Model Construction. **(A)** Differential gene analysis was performed to identify genes that differ between the normal and tumor groups. **(B)** Cox regression analysis was conducted on genes associated with cellular senescence. **(C)** The intersection of differential genes and prognostic genes was extracted. **(D)** The correlation between nine differentially expressed prognostic genes was analyzed and visualized using a correlation circle plot. **(E)** A prognostic prediction model was constructed using the LASSO algorithm. **(F)** The optimal number of variables was determined based on the λ value.


Through differential gene expression analysis, we identified significantly upregulated (red) and downregulated (green) DEGs in tumor samples, as visualized in the volcano plot (**Figure 1A**). To ascertain 21 CSAGs that impact the prognosis of THCA, a univariate Cox regression analysis was executed (p < 0.05, HR ≠ 1, **Figure 1B**). By intersecting the 61 DEGs with the 21 prognostic genes, we identified 9 genes that were present in both gene sets (**Figure 1C**). These genes were: HDAC4, NDRG1, NEK1, NINJ1, PLA2R1, SNAI1, ASPH, CDKN2A, and E2F1. An analysis of the correlation network for these 9 genes showed that HDAC4, NDRG1, NEK1, NINJ1, PLA2R1, SNAI1, and ASPH exhibited predominantly positive correlations amongst themselves. Additionally, CDKN2A and E2F1 displayed a positive correlation with each other. However, the expression levels of these genes were inversely related to the majority of the other genes in the network (**Figure 1D**). Taking these observations into account, we refined the gene set further and developed a prognostic model employing the LASSO algorithm. The coefficient path distribution for the 9 genes showed that as log(λ) increased, the coefficients of the genes gradually approached zero in a stepwise manner (**Figure 1E**). The optimal number of genes, determined when the cross-validation curve reached its minimum, corresponding to the lowest partial likelihood deviance, was found to be 6 genes (**Figure 1F**). The model equation is as follows:

Risk score= ASPH*0.236612398528079 + CDKN2A*0.55903013908233 + E2F1*(−0.429431075541726) + DRG1*0.455159278181108 + NINJ1*(−0.555409521362013) + SNAI1*1.1381189402696

**Figure 2:**
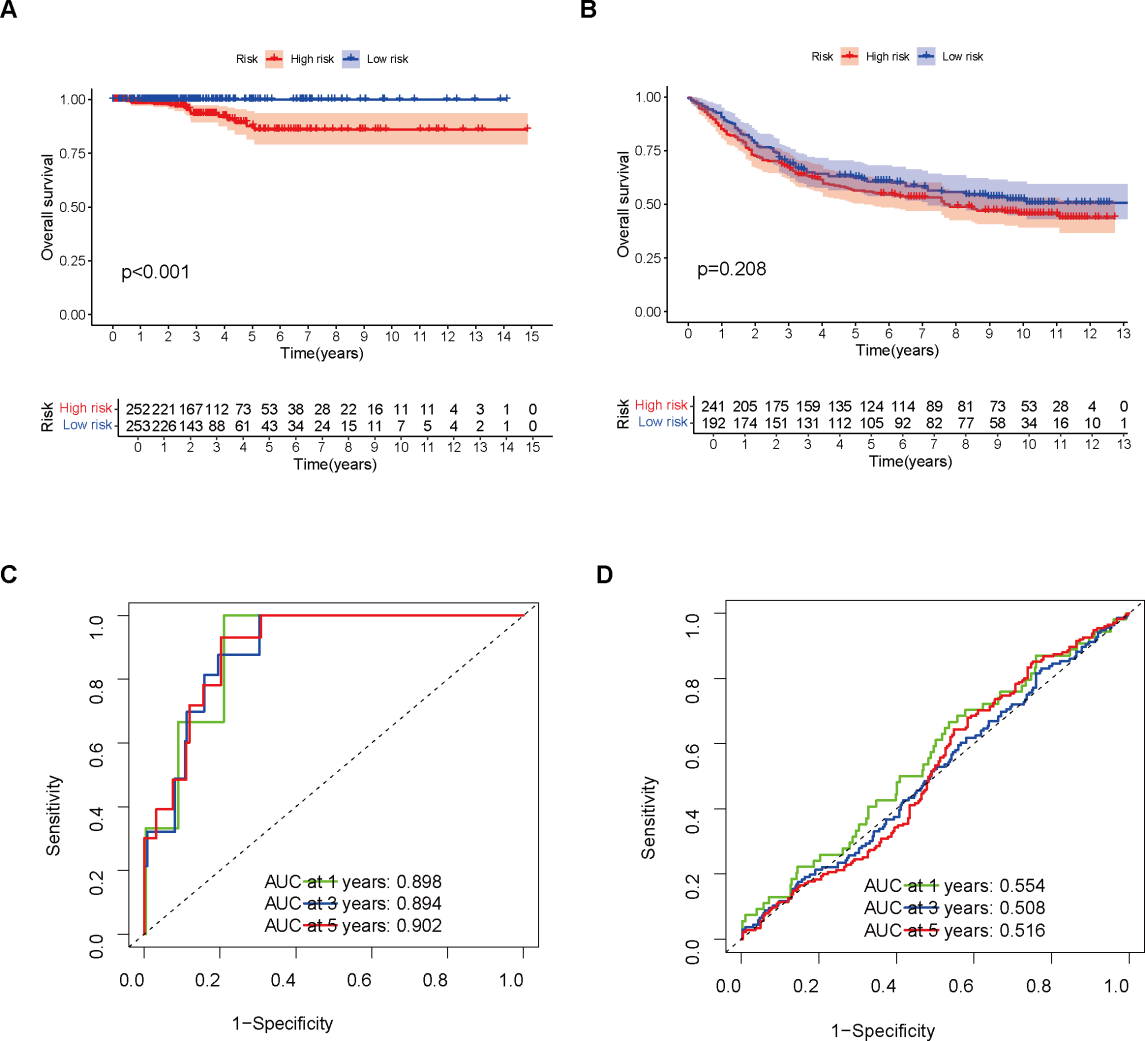
Model Validation Using Training and Validation Sets. **(A)** Survival analysis was performed on the training set. **(B)** Survival analysis was conducted on the validation set. **(C)** The model’s performance in the training set was evaluated using a ROC curve. **(D)** The model’s performance in the validation set was assessed using a ROC curve.

In comparison to the high-risk group, the TCGA cohort’s low-risk patient group demonstrated a notably superior OS outcome (p < 0.001, **Figure 2A**). The prognostic difference between the two groups in the GEO cohort was further validated by us (**Figure 2B**). Furthermore, by analyzing the ROC curves associated with 1-year, 3-year, and 5-year survival rates, it was demonstrated that the model demonstrated robust diagnostic capabilities in both independent patient groups (**Figures 2C, D**).

### 3.2 Enrichment and prognostic analysis conducted for various risk groups

**Figure 3:**
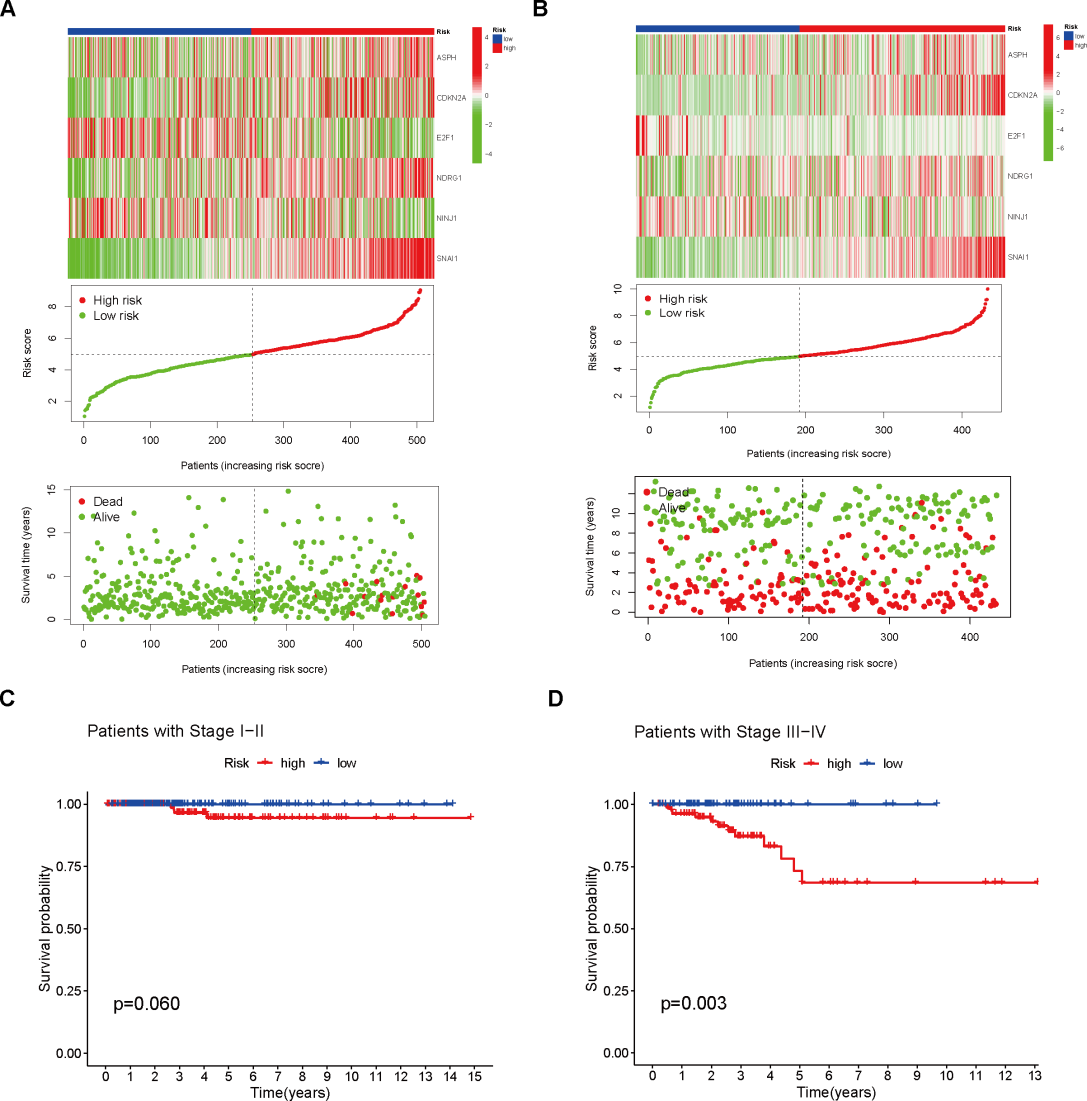
Analysis of Different Risk Groups. **(A)** Risk scores were calculated and the training set was divided into high- and low-risk groups. Heatmaps were used to visualize the differential model genes between the two risk groups. The cumulative risk factor plot illustrates the changes in patient survival time and status with respect to the risk score. **(B)** Risk scores were calculated and the validation set was divided into high- and low-risk groups. Heatmaps were used to visualize the differential model genes between the two groups. The cumulative risk factor plot illustrates the changes in patient survival time and status with respect to the risk score. **(C)** Survival analysis was performed on the high- and low-risk groups of stage I and II patients. **(D)** Survival analysis was performed on the high- and low-risk groups of stage III and IV patients.

**Figure 4:**
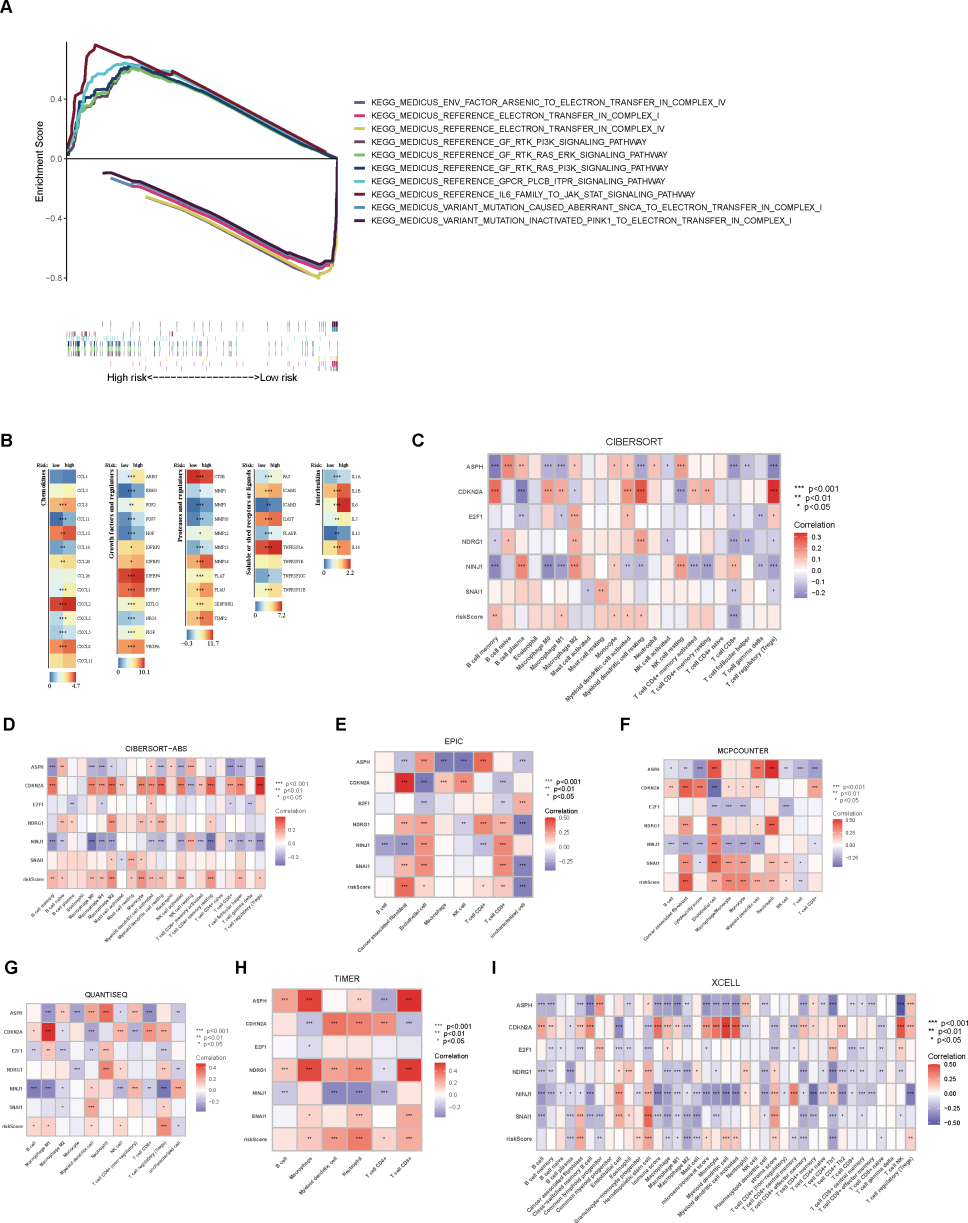
Enrichment analysis and immune characteristic differential analysis of high- and low-risk groups. **(A)** GSEA enrichment analysis of the high- and low-risk groups. **(B)** Differential expression analysis of immune regulatory genes between the two groups. **(C)** CIBERSORT analysis of the correlation between immune cell scores and model genes/risk score. **(D–I)** Analysis of immune cell scores and their correlation with model genes/risk score in the samples using CIBERSORT-ABS, EPIC, MCPCOUNTER, QUANTISEQ, TIMER, and XCELL algorithms.

An analysis of THCA samples focusing on the expression levels of six model genes (ASPH, CDKN2A, E2F1, NDRG1, NINJ1, SNAI1) revealed that those who exhibited high expression of ASPH, CDKN2A, NDRG1, and SNAI1 were predominantly grouped into the high-risk category. In contrast, those who showed elevated expression of both E2F1 and NINJ1 were mainly classified into the low-risk group. Additionally, a higher percentage of patients in the low-risk group were found to be 5-year survivors (**Figures 3A, B**). Further analysis of KM survival curves for TCGA-THCA patients with tumor stages I-II revealed no statistically significant difference in survival probabilities between the two risk groups (p = 0.060, **Figure 3C**). For patients in stages III-IV, the risk groups showed a more distinct difference in prognosis, with the high-risk group having a significantly lower survival probability than the low-risk group (p = 0.003, **Figure 3D**). In addition, through GSEA analysis, it was found that genes in the high-risk group showed considerable enrichment in the pathways related to the mitochondrial electron transport chain, among which the following five pathways related to the electron transport process in the mitochondrial respiratory chain in the KEGG MEDICUS database had the highest enrichment: ENV_FACTOR_ARSENIC_TO_ELECTRON_TRANSFER_IN_COMPLEX_IV, REFERENCE_ELECTRON_TRANSFER_IN_COMPLEX_I, REFERENCE_ELECTRON_TRANSFER_IN_COMPLEX_IV, VARIANT_MUTATION_CAUSED_ABERRANT_SNCA_TO_ELECTRON_TRANSFER_IN_COMPLEX_I, VARIANT_MUTATION_INACTIVATED_PINK1_TO_ELECTRON_TRANSFER_IN_COMPLEX_I. Conversely, the low-risk group genes exhibited significant enrichment in pathways related to cell proliferation, survival, and metabolic regulation, especially within the five most enriched pathways listed in the KEGG MEDICUS database: REFERENCE_GF_RTK_PI3K_SIGNALING_PATHWAY, REFERENCE_GF_RTK_RAS_ERK_SIGNALING_PATHWAY, REFERENCE_GF_RTK_RAS_PI3K_SIGNALING_PATHWAY, REFERENCE_GPCR_PLCB_ITPR_SIGNALING_PATHWAY, REFERENCE_IL6_FAMILY_TO_JAK_STAT_SIGNALING_PATHWAY (**Figure 4A**).

### 3.3 Differential immune characteristics analysis

Upon examining the heatmap depicting the differential expression levels of immune modulators across various risk groups, it was evident that the high-risk group demonstrated heightened activity of five immune regulatory molecules (p < 0.05, **Figure 4B**). Our results, utilizing the CIBERSORT algorithm, revealed a positive correlation between the abundance of several immune cell types and the risk score, particularly memory B cells, M1 macrophages, monocytes, activated myeloid dendritic cells, and resting myeloid dendritic cells. Conversely, a significant negative association was observed for CD8+ T cells. Additionally, a significant negative relationship was noted between both CD8+ T cells and regulatory T cells (Tregs) and over half of the model’s genes. Among the six model genes, ASPH, CDKN2A, and NINJ1 exhibited strong correlations with tumor-associated immune cells. In particular, the expression level of CDKN2A exhibited a positive link with the level of immune cell presence, whereas NINJ1 showed a negative correlation with immune cells at the expression level (p < 0.05, **Figure 4C**). Employing various algorithms yielded consistent results, suggesting that, apart from the general positive link between risk score and immune cell abundance, a substantial number of model genes exhibited notable correlations with the levels of immune cell infiltration. Notably, CDKN2A exhibited a stronger positive correlation with immune cells compared to the other genes, while NINJ1 showed a more pronounced negative relationship with immune cells (p < 0.05, **Figures 4D–I**).

**Figure 5:**
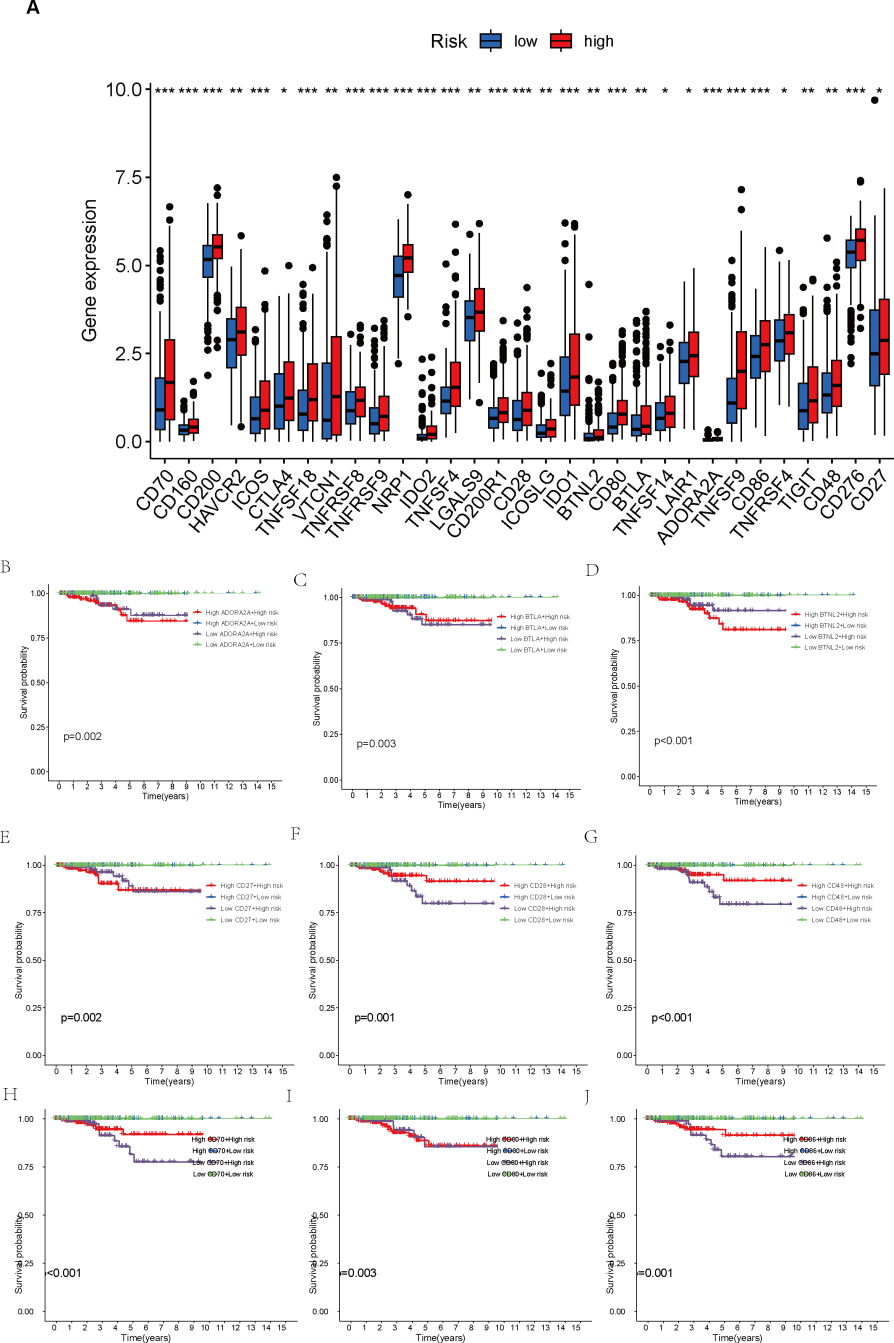
Comparison of immune checkpoint expression levels and their impact on prognosis between high- and low-risk groups. **(A)** Comparison of expression levels of 31 immune checkpoint genes between the high- and low-risk groups. **(B–J)** Survival analysis of high- and low-risk groups stratified by immune checkpoint gene expression levels. *p < 0.05; **p < 0.01; ***p < 0.001.

**Figure 6:**
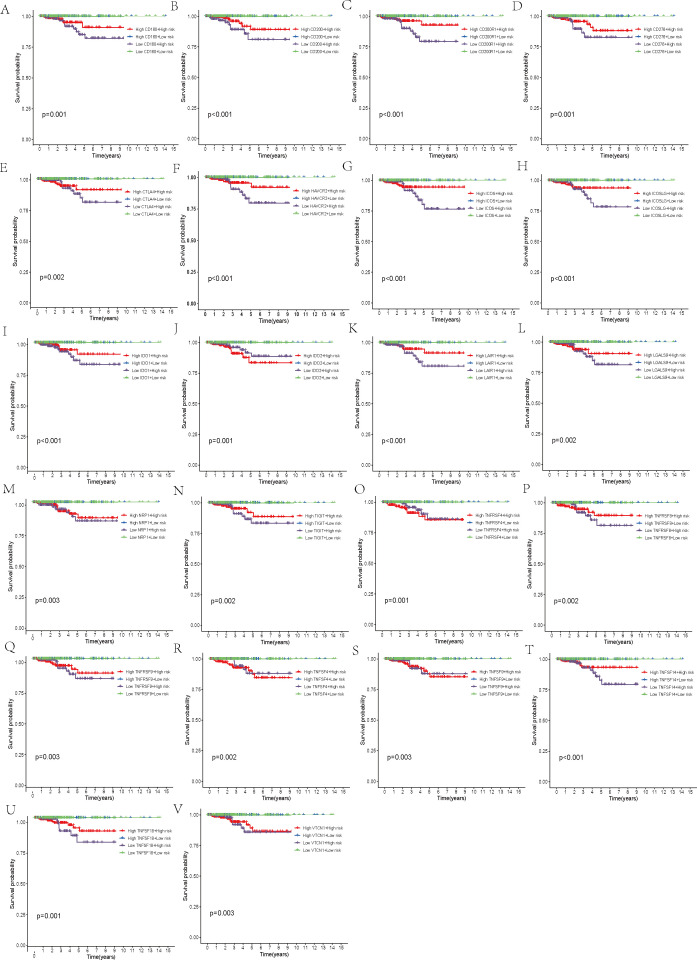
Survival analysis. **(A–V)** Stratification of the high- and low-risk groups based on immune checkpoint gene expression levels.

Furthermore, a comparison of the expression profiles of 31 immune checkpoint genes was conducted between the two risk groups. Boxplot analysis revealed that all immune checkpoint genes were significantly upregulated in the high-risk group (p < 0.05, **Figure 5A**), suggesting that high expression of immune checkpoints might be associated with unfavorable tumor prognosis. To further explore the impact of different immune checkpoint gene expressions on patient prognosis, we performed KM analysis. Survival curves for different subgroups indicated that, regardless of whether immune checkpoint genes were highly expressed, samples with higher risk scores consistently showed significantly lower survival probabilities compared to those with lower risk scores. The prognostic model demonstrates a strong capacity for prediction, underlining its robustness. Certain immune checkpoint genes, when upregulated in the high-risk group, showed a degree of association with improved patient prognosis. Specifically, higher expression levels of BTLA, CD28, CD48, CD70, CD86, CD160, CD200, CD200R1, CD276, CTLA4, HAVCR2, ICOS, ICOSLG, IDO1, LAIR1, LGALS9, NRP1, TIGIT, TNFRSF8, TNFRSF9, TNFSF14, TNFSF18, and VTCN1 were associated with better prognosis. Patients exhibiting high expression of ADORA2A, BTNL2, CD27, CD80, IDO2, TNFRSF4, TNFSF4, and TNFSF9 had a worse prognosis in comparison to those with low expression levels, conversely (p < 0.01, **Figures 5B–J**, **6A–V**).

### 3.4 Mutation analysis and survival analysis of TMB

**Figure 7:**
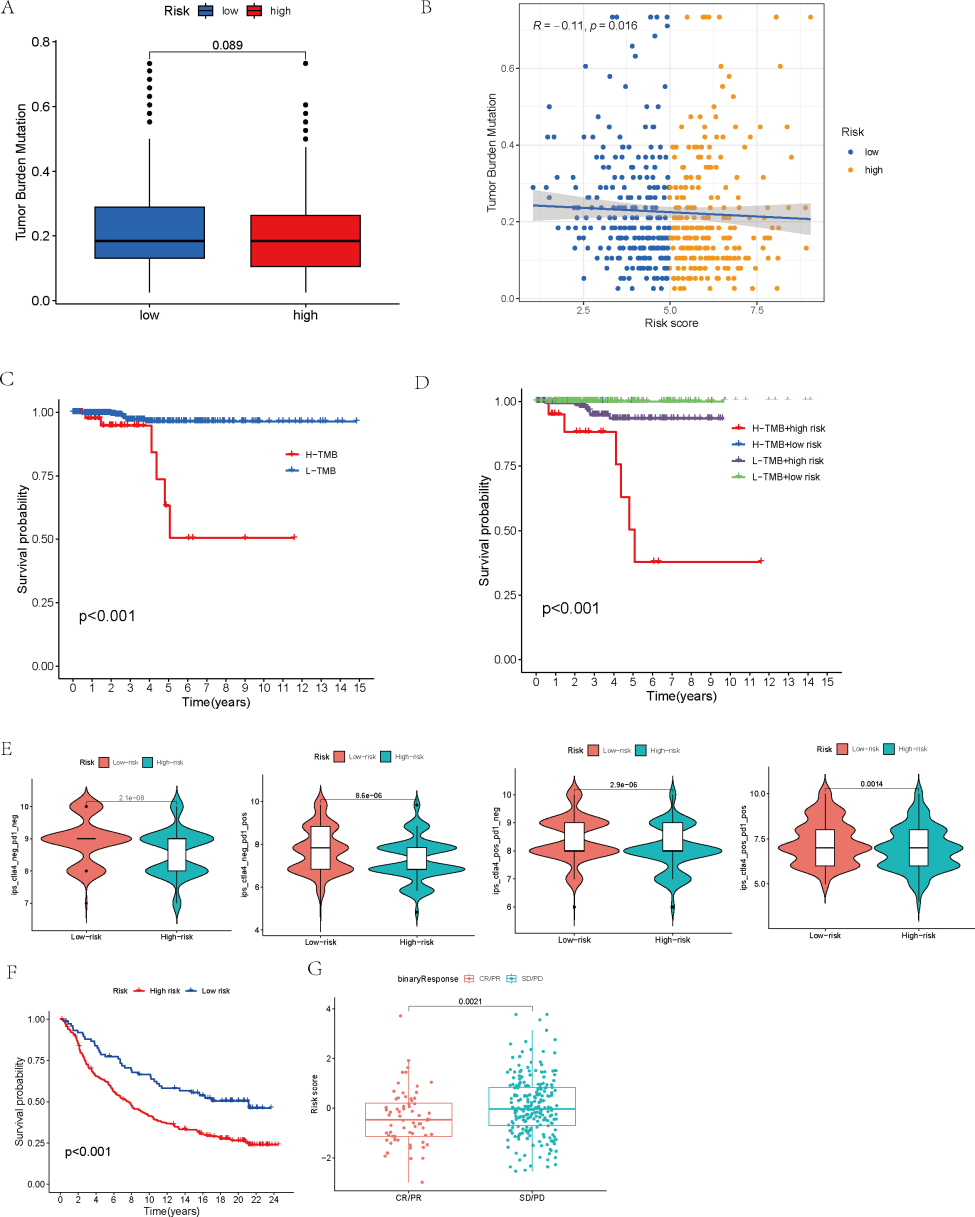
Mutation analysis and survival analysis based on TMB, and prediction of tumor treatment responses by the model. **(A)** Tumor mutation burden analysis of the high- and low-risk groups. **(B)** Analysis of the correlation between tumor mutation burden and risk score. **(C)** Survival analysis of high-TMB and low-TMB groups. **(D)** Survival analysis of high- and low-risk groups within high- and low-TMB subsets. **(E)** Analysis of immune treatment responses in high- and low-risk groups. **(F)** Survival analysis of high- and low-risk groups in the IMvigor210 immunotherapy cohort. **(G)** Analysis of risk model score differences between disease status groups in the IMvigor210 immunotherapy cohort.

A comparison of TMB between the high-risk and low-risk groups revealed no statistically significant variation between the two (p = 0.089, **Figure 7A**). Nevertheless, additional correlation analysis unveiled an inverse relationship between TMB and risk score, where an increase in risk score was accompanied by a decrease in TMB (R = -0.11, p = 0.016, **Figure 7B**). After stratifying patients by their TMB and analyzing the survival curves of the H-TMB and L-TMB groups, we found the H-TMB group had a notably lower survival rate than the L-TMB group, which indicates that there may be a potential association between higher TMB and a poorer prognosis (p < 0.001, **Figure 7C**). In order to delve deeper into how both TMB and risk score collectively influence the prognosis of THCA, we performed a KM survival analysis incorporating the risk score. On one hand, high-risk scores corresponded to lower survival probabilities. Conversely, while the prognosis of low-risk patients remained relatively unaffected by TMB, high-risk patients who also had high TMB demonstrated significantly diminished survival rates in comparison to their counterparts with low TMB within the group with elevated risk (p < 0.001, **Figure 7D**).

### 3.5 The model’s capacity to predict tumor treatment outcomes

Moreover, after conducting an analysis of the IPS across distinct risk groups, specifically ips_ctla4_neg_pd1_neg, ips_ctla4_neg_pd1_pos, ips_ctla4_pos_pd1_neg, and ips_ctla4_pos_pd1_pos, it became evident that the IPS within the low-risk group surpassed those in the other categories, suggesting a superior responsiveness of the low-risk group to both CTLA-4 and PD-1 inhibitors, especially in scenarios involving monotherapy with PD-1 inhibitors (p < 0.01, **Figure 7E**).

**Figure 8:**
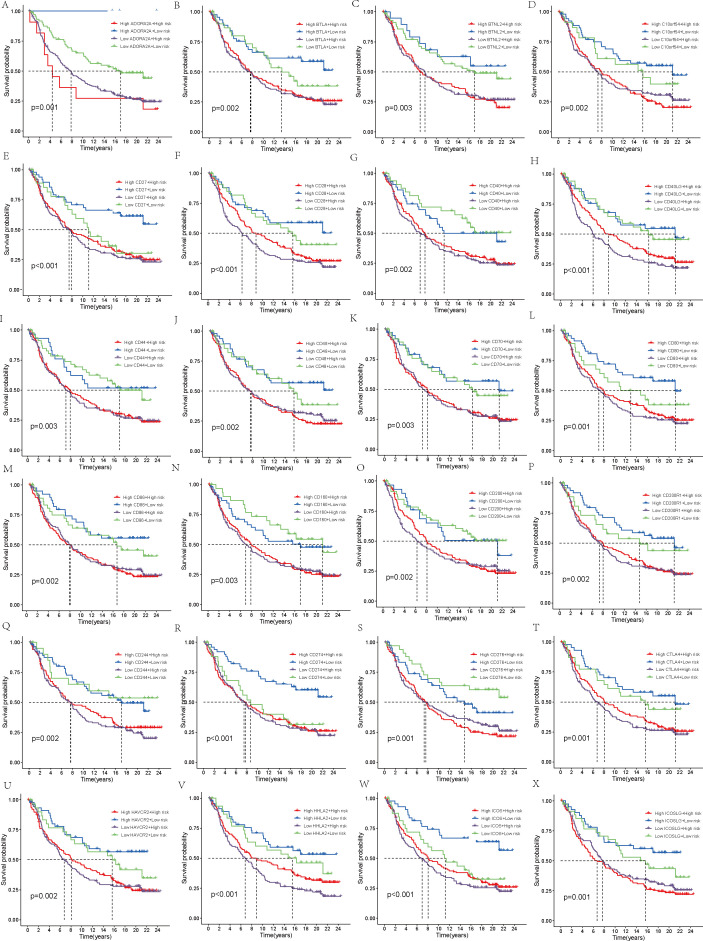
Survival analysis. **(A–X)** In the IMvigor210 cohort, the high-risk and low-risk groups were stratified according to the level of immune checkpoint gene expression and the difference in prognosis was compared.

**Figure 9:**
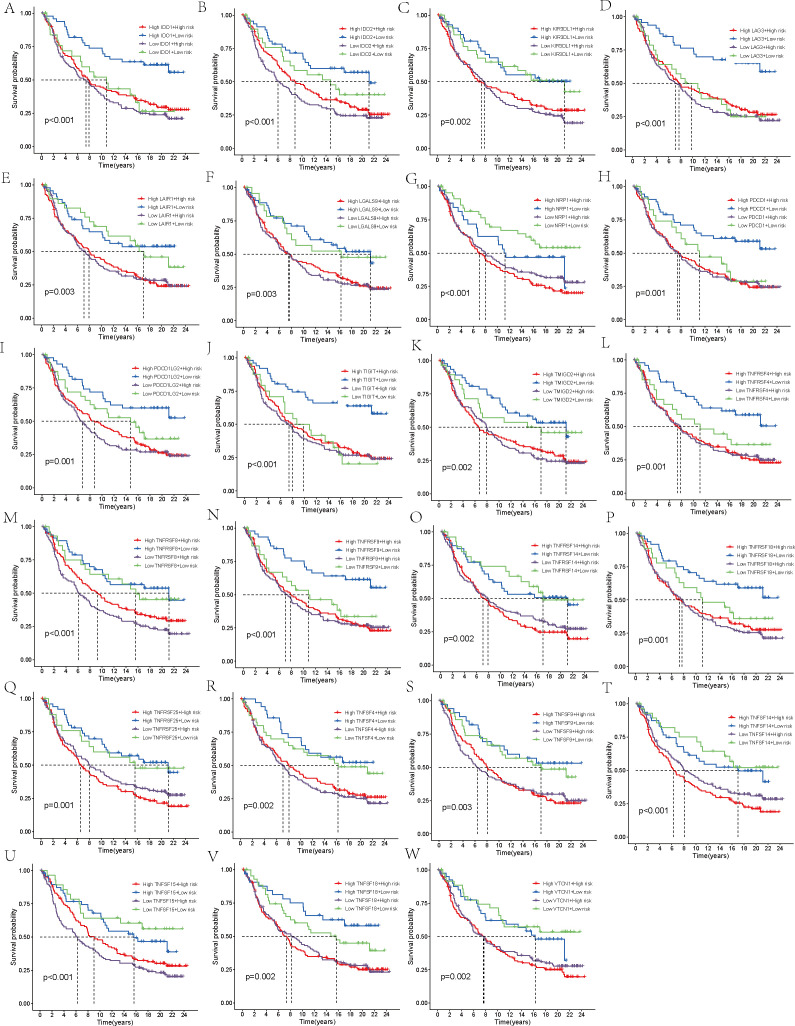
Survival analysis. **(A–W)** In the IMvigor210 cohort, the high-risk and low-risk groups were stratified according to the level of immune checkpoint gene expression and the difference in prognosis was compared.

Finally, the IMvigor210 immunotherapy cohort was used to validate the model. The survival curves generated by the Kaplan-Meier method for the two risk groups within the IMvigor210 dataset revealed that high-risk samples exhibited a worse prognosis compared to low-risk samples, thereby reinforcing the model’s capacity for generalization (p < 0.01, **Figure 7F**). Moreover, the predictive capability of the risk model concerning chemotherapy response was assessed, revealing that patients in the CR/PR category had notably lower risk scores compared to those in the SD/PD category (p = 0.0021, **Figure 7G**). Based on these findings, we propose that the risk model may serve as a reliable predictive tool for treatment response in patients with THCA. Additionally, we broadened our analysis, which was aimed at gauging the effect of the immune checkpoint co-modeling on the prognosis within the IMvigor210 dataset. In general, irrespective of the expression levels of immune checkpoint genes, patients categorized in the low-risk group exhibited notably superior survival outcomes compared to those in the high-risk group. Specifically, the upregulation of genes including CD40, CD200, CD244, CD276, NRP1, TNFRSF14, TNFSF14, TNFSF15, and VTCN1 was associated with a significant enhancement in the survival probability of patients belonging to the low-risk group. In contrast, when genes like BTLA, CD27, CD28, CD40, CD40LG, CD80, CD244, CD274, CTLA4, HHLA2, ICOS, IDO1, IDO2, KIR3DL1, LAG3, TNFRSF8, TNFRSF18, and TNFSF15 were highly expressed, high-risk patients exhibited significantly improved prognosis (p < 0.01, **Figures 8**, **9**).

## 4 Discussion

Human THCA stands as the most frequent endocrine tumor and ranks seventh among cancers most commonly diagnosed in women (26). Over the past few decades, there has been a consistent rise in its incidence, resulting in a current prevalence that constitutes 3-4% of all cancer cases (1). Although the prognosis for most THCA patients is favorable, early detection and diagnosis remain challenging (8). Once tumor cells metastasize to distant sites, survival rates vary significantly depending on the pathological subtype (10), with the median survival for ATC often limited to only 3-6 months (11). Worse still, the standard therapies, such as surgery and postoperative radioiodine ablation, are ineffective for ATC patients (9). Moreover, targeted therapies and immunotherapies have limited success in achieving curative outcomes for certain DTC and MTC patients (12). Therefore, the objective of this study extends beyond merely exploring the influence of cellular senescence on THCA and its fundamental mechanisms, but also to develop a prognostic prediction model, with the goal of identifying novel and effective therapeutic targets to improve the prognosis and therapeutic outcomes for THCA patients.

Initially, a detailed examination of CSAG variations between normal and tumor tissues was carried out, resulting in the discovery of 61 DEGs. Subsequently, we conducted a Cox regression analysis of the CSAG, which yielded 21 prognostic-associated aging-related genes. By intersecting these 21 prognostic genes with the 61 DEGs, we identified 9 DEGs that were associated with prognosis. The genes that overlap may offer significant understanding of CSAG’s role in predicting the outcome of THCA and might be candidates for new prognosis prediction targets and therapeutic approaches. However, Nonetheless, there is a scarcity of research investigating the connection between these genes and THCA, and the interactions between these genes remain unclear. Therefore, we analyzed the correlations among the 9 genes and found that HDAC4, NDRG1, NEK1, PLA2R1, and ASPH showed strong positive correlations with other genes, with ASPH, HDAC4, and NEK1 demonstrating particularly strong associations. It is known that HDAC4 promotes carcinogenesis by limiting the transcription of tumor suppressor genes (27), while NEK1 is involved in DNA damage repair (28). Although CDKN2A and E2F1 are positively correlated, they show negative correlations with the expression levels of most other genes. Next, we used LASSO to perform gene selection for model construction in the training set, ultimately identifying six model genes: ASPH, CDKN2A, E2F1, NDRG1, NINJ1, and SNAI1. The transmembrane protein Aspartate β-hydroxylase (ASPH), weighing approximately 86 kDa and belonging to the highly conserved α-ketoglutarate-dependent dioxygenase family, is classified as a type II protein. ASPH has been found to be overexpressed in various malignant tumors (29), and The hydroxylase activity it possesses holds a crucial function in fostering malignant tumor characteristics, encompassing tumor growth, proliferation, invasion, and metastasis. Research has shown that ASPH not only influences the prognosis of hepatocellular carcinoma under the regulation of inositol polyphosphate-5-phosphatase F (INPP5F) (30), but also promotes tumor progression and poor prognosis by activating Notch and PI3K-dependent signaling pathways, inducing a delay in tumor cell senescence and impairing mitochondrial integrity (31). One of the most frequently deleted homozygous genes in human cancers is CDKN2A, situated on chromosome 9 (32). The tumor suppressors p16 and p14arf are both products of CDKN2A (33, 34). Since p16 inhibits the G1 to S phase transition and p14arf activates the tumor suppressor p53 (34, 35), the loss of CDKN2A function leads to cell cycle dysregulation and promotes tumor development. E2F transcription factor 1 (E2F1) is the archetype member of the E2F family, which includes transcriptional activators that bind to the adenoviral E2 promoter (36). In regulating the expression of a multitude of oncogenes and tumor suppressor genes, E2F1 serves as an activator. The E2F family precisely regulates the cell cycle, apoptosis, and DNA replication processes (37). E2F1 not only promotes cell migration and metastasis but also plays a critical role in stem cell-mediated carcinogenesis and estrogen-mediated cell proliferation (38). Its non-transcriptional activities further promote DNA repair or induce autophagy and apoptosis (39). N-myc downstream regulated gene 1 (NDRG1), a gene that functions to suppress tumorigenesis, located on chromosome 8q24.3, encodes a 3.0 kb mRNA and inhibits cell proliferation, migration, invasion, and autophagy, while promoting apoptosis and differentiation, thus suppressing tumor invasive phenotypes (40). Overexpression of NDRG1 downregulates cyclin D1, a Wnt-responsive gene, and inhibits cell cycle progression (41). Although NDRG1 primarily exhibits anti-cancer and anti-metastasis functions, it has also been shown to promote cancer in certain cancers such as gastric cancer and hepatocellular carcinoma (42). Therefore, some researchers suggest that NDRG1 may exert pleiotropic effects depending on the cancer type (43). Initially discovered as a gene that undergoes significant upregulation in Schwann cells and dorsal root ganglia following nerve injury, Ninjurin1 (NINJ1) serves as a homophilic cell adhesion molecule (CAM) (44). The regulation of neovascularization *in vitro* and the formation of the hyaloid vascular system *in vivo* are mechanisms through which NINJ1 contributes to angiogenesis, and NINJ1 forms a feedback loop with p53, whereby NINJ1, as a p53 target, suppresses p53 mRNA translation. Moreover, NINJ1 exerts opposite effects on cell growth, migration, and tumor development through wild-type and mutant p53 (45). Additionally, NINJ1 inhibits the IL-6 signaling pathway both *in vitro* and *in vivo*, suppressing lung cancer migration, invasion, and metastasis (46). Snail family zinc finger 1 (SNAI1) is the first and most extensively studied E-cadherin transcriptional repressor, and E-cadherin, encoded by the epithelial gene CDH1, is a marker of epithelial-mesenchymal transition (EMT), a developmental process that cancer cells use to promote invasion, metastasis, and therapy resistance (47). In normal tissues, the regulation of SNAI1 expression is precise, whereas its deregulation is linked to the advancement of several types of cancer (48, 49). In addition to repressing the E-cadherin gene, the core function of SNAI1 includes the transcriptional repression of tight junction genes and fructose-1,6-bisphosphatase genes, which regulate glycolysis rate (50). In ovarian cancer cells, SNAI1 primarily regulates intercellular and cell-matrix adhesion (51). We built a model for predicting prognosis by utilizing six model genes, and subsequently assessed the scores for patients in not only the training but also the validation datasets. Following that, the patients from both cohorts were divided into two categories – high-risk and low-risk – according to their respective risk scores. An analysis comparing the survival rates of the two groups within both cohorts unveiled that patients in the low-risk category of the TCGA cohort exhibited a notably superior prognosis compared to those in the high-risk category. Additionally, we assessed the model’s predictive power by employing ROC curves and found that it demonstrated high accuracy in both the two datasets. Given the roles of these six genes in malignant tumors, we propose that they are likely to serve as prognostic biomarkers for THCA and may influence the initiation and progression of THCA. An additional analysis was conducted across two datasets to explore the variations in the expression of model genes across the cohorts stratified as high-risk and low-risk. It is apparent that ASPH, CDKN2A, NDRG1, and SNAI1 demonstrated increased expression in the high-risk group, irrespective of whether they were in the training set or the validation set. In the low-risk group, E2F1 and NINJ1 were more expressed. An analysis was conducted to compare the survival differences between the two groups, revealing that, although the high-risk group had a lower survival rate, the maximum survival duration of patients did not significantly differ from that of the low-risk group. We then conducted survival analysis for the TCGA cohort, stratifying patients into high-risk and low-risk groups based on Stage I-II and Stage III-IV, in order to investigate how tumor stage influences the predictive ability of the model. Comparable survival rates were observed between the high- and low-risk groups in Stage I-II, with no statistically significant differences emerging. However, in Stage III-IV, a notable disparity in survival rates was observed between the two groups. We interpret this as suggesting that our model holds greater predictive value in patient populations with more advanced stages. Additionally, an analysis using GSEA, focusing on the KEGG MEDICUS pathway, was conducted, revealing the enrichment of five pathways, primarily associated with cellular proliferation, differentiation, and signaling, for those at high risk. Genes in the high-risk group were highly enriched in pathways associated with the mitochondrial electron transport chain, reflecting changes in cellular energy metabolism in the high-risk group and increased apoptosis that may result from mitochondrial dysfunction. Conversely, for those at low risk, the primary association of the enriched pathways was with mitochondrial electron transport and oxidative phosphorylation processes. The results of our study introduce novel understandings into the realm of THCA treatment, implying that a customized exploration of therapeutic options for patients stratified into high- and low-risk groups may facilitate the development of more precise targeted therapies.

Following that, an analysis of the immune regulatory expression profiles was conducted for both groups, with the results showing that the high-risk group had significantly increased expression levels of five immune regulatory molecules compared to the low-risk group. This suggests that the response to immune checkpoint inhibitors or immunotherapies in THCA may differ based on the risk scores. This also indicates that the patient’s immune status is strongly associated with clinical outcomes. Afterwards, various algorithms were employed under the purpose of examining the relationship existing between the abundance of immune cell infiltration and six model genes. The analysis revealed a positive association between the risk score and CDKN2A, both related to increased immune cell abundance, whereas NINJ1 displayed an inverse relationship with the expression levels of immune cells. The indication is that CDKN2A and NINJ1 potentially impact tumor prognosis by regulating the infiltration of immune cells. We subsequently conducted an analysis to assess the variability in the expression of immune checkpoints across patient groups stratified by high and low risk. The results of our study indicated that there was an upregulation of 31 immune checkpoints among those in the high-risk category, hinting at a potentially more favorable efficacy of immune checkpoint inhibitors in this group. By utilizing the median expression levels of these 31 immune checkpoints, we further divided both high- and low-risk groups into two subgroups and conducted survival analysis for each checkpoint. Despite immune checkpoint expression having little effect on the survival of patients in the low-risk group, the high-risk group exhibited a notable correlation between immune checkpoint expression and their survival rates. Eight immune checkpoints—ADORA2A, BTNL2, CD27, CD80, IDO2, TNFRSF4, TNFSF4, and TNFSF9—were associated with poorer prognosis when highly expressed, while the high expression of most immune checkpoints was generally linked to better outcomes. This finding provides novel insights into the development of novel immunotherapy agents targeting immune checkpoints, and indicates that the level of immune checkpoint expression may serve as a marker for evaluating disease progression and prognosis, thereby laying the groundwork for tailored treatment approaches.

After computing and contrasting the TMB of the two patient groups, we observed no notable disparity, which could be attributed to the influence of potential confounding factors. The Pearson correlation analysis was conducted by us to deeply evaluate the correlation between the risk score and TMB, with the aim of bolstering the credibility of our findings. The results of our analysis revealed an inverse relationship between the risk score and TMB, implying that patients with higher risk scores had correspondingly lower TMB levels. With patients categorized into H-TMB and L-TMB groups using the median TMB value as a cutoff, we proceeded to analyze their survival rates. Our analysis revealed that patients belonging to the H-TMB group had significantly diminished survival rates in comparison to those in the L-TMB group. Subsequently, we combined TMB and risk scores to classify the patients into four subgroups for survival analysis. Our results showed that, although TMB had no substantial effect on survival among patients in the low-risk group, patients in the high-risk group with high TMB had significantly inferior survival rates compared to those with low TMB. This finding supports our previous conclusion that TMB is not an independent prognostic factor. TMB levels appear to influence the prognosis primarily in high-risk patients. Although high-risk groups generally correspond to lower TMB, patients within these groups who have higher TMB tend to experience poorer outcomes. Some studies suggest that a higher TMB reflects greater exposure to tumor antigens, and thus TMB could potentially serve as a marker for the response to therapy utilizing immune checkpoint inhibitors (52). A deeper exploration into the function of TMB in THCA is necessary, as it could potentially present a new method for therapeutic intervention and prognosis assessment in THCA.

Finally, we conducted an immunotherapy analysis on the patients, separately assessing high-risk and low-risk patients’ reactions to anti-CTLA-4 and anti-PD-L1 antibodies. This led to the identification of four distinct IPS. Our results suggest that, regardless of whether patients received anti-CTLA-4 or anti-PD-L1 antibodies, those in the low-risk group responded significantly better to immune checkpoint inhibitor treatment strategies. Notably, patients receiving monotherapy with PD-1 inhibitors exhibited the most pronounced difference. Our model suggests that it can direct the choice of more precisely targeted immunotherapy strategies according to a patient’s risk score, which may lead to an enhanced response to immune suppressors for high-risk patients and, consequently, a better prognosis. To further assess the predictive efficacy of the model, we used the IMvigor210 cohort for validation. After determining the risk scores for the patients, the IMvigor210 cohort was categorized into those at high risk and those at low risk, upon which survival analysis was subsequently performed. The predictive performance of the model was validated by the results as being robust. We then classified patients into two groups based on therapeutic response: complete or partial response (CR/PR) and stable or progressive disease (SD/PD), and compared their risk scores. The CR/PR group exhibited significantly decreased risk scores when compared to the SD/PD group. Subsequently, the patients’ risk scores were combined with the expression levels of 48 immune checkpoint molecules, leading to the classification of patients into four subgroups, each characterized by the expression pattern of a particular immune checkpoint molecule. The analysis of survival outcomes showed that, when compared to patients in the high-risk group, those in the low-risk group exhibited a remarkably better prognosis. Within the low-risk group, there was an upregulation of specific immune checkpoint genes, such as CD40, CD200, CD244, CD276, NRP1, TNFRSF14, TNFSF14, TNFSF15, and VTCN1, was linked to a notable elevation in the likelihood of survival, suggesting that these genes may serve as promising therapeutic targets for low-risk patients. Other studies have also linked these genes to thyroid cancer (53, 54). When it comes to the group with higher risk scores, over one-third of the immune checkpoint genes studied were identified as having a positive correlation with a better prognosis. Genes like CD40, CD244, and TNFSF15 were found to be beneficial for the prognosis of both groups. These findings open up new possibilities for targeted therapies in THCA.

While our study established a prognostic model for THCA and uncovered the role of CSAGs, limitations exist. First, using public database data may introduce sample bias. Additionally, findings are primarily data-driven, lacking experimental validation. Lastly, analysis of immune checkpoints and TMB was limited to risk stratification, requiring further investigation into their mechanisms.

## 5 Conclusion

In this study, we used bioinformatics to explore cellular senescence’s impact on THCA prognosis. By integrating public database data and focusing on CSAGs, we developed a robust prognostic model validated by KM and ROC curves. By stratifying patients into high- and low-risk groups, the model uncovered notable disparities in prognosis, immune activity, and treatment response. Risk-stratified analysis provided insights into immune checkpoints and TMB. Our findings deepen understanding of cellular senescence in THCA and suggest new therapeutic targets.

## Data Availability

The original contributions presented in the study are included in the article/**Supplementary Material**. Further inquiries can be directed to the corresponding author.
